# Prospective evaluation of a telmisartan suppression test as a diagnostic tool for primary hyperaldosteronism in cats

**DOI:** 10.1111/jvim.16741

**Published:** 2023-05-29

**Authors:** Maxime Kurtz, Virginie Fabrès, Renaud Dumont, Valérie Chetboul, Sabine Chahory, Vittorio Saponaro, Emilie Trehiou, Camille Poissonnier, Peggy Passavin, Coline Jondeau, Matthieu Bott, Thierry Buronfosse, Ghita Benchekroun

**Affiliations:** ^1^ Ecole Nationale Vétérinaire d'Alfort – CHUVA, Service de Médecine Interne Maisons‐Alfort France; ^2^ Aquivet Clinique Veterinaire – Service de Médecine Interne Eysines France; ^3^ Centre Hospitalier Veterinaire Fregis Arcueil France; ^4^ École Nationale Vétérinaire d'Alfort – CHUVA, Unité de Cardiologie d'Alfort Maisons‐Alfort France; ^5^ Ecole Nationale Vétérinaire d'Alfort – CHUVA, Unité d'Ophtalmologie Maisons‐Alfort France; ^6^ VetAgro Sup – Service De Biologie Clinique Marcy‐l'Etoile France; ^7^ Ecole Nationale Vétérinaire d'Alfort – University Paris‐Est Créteil, INSERM, IMRB Maisons‐Alfort France

**Keywords:** adrenal, adrenal gland, aldosterone, cardiology, cardiovascular, endocrinology, hemodynamics, hypertension, hypertrophic cardiomyopathy, hypokalemia, retinopathy

## Abstract

**Background:**

In a previous study, telmisartan suppressed aldosterone secretion in healthy cats but not in cats with primary hyperaldosteronism (PHA).

**Hypotheses:**

Telmisartan suppresses aldosterone secretion in middle‐aged healthy cat and cats with diseases that may result in secondary hyperaldosteronism, but not in those with PHA.

**Animals:**

Thirty‐eight cats: 5 with PHA; 16 with chronic kidney disease (CKD), subclassified as hypertensive (CKD‐H) or non‐hypertensive (CKD‐NH); 9 with hyperthyroidism (HTH); 2 with idiopathic systemic arterial hypertension (ISH); and 6 healthy middle‐aged cats.

**Methods:**

Prospective, cross‐sectional study. Serum aldosterone concentration, potassium concentration, and systolic blood pressure were measured before and 1 and 1.5 hours after PO administration of 2 mg/kg of telmisartan. The aldosterone variation rate (AVR) was calculated for each cat.

**Results:**

No significant difference in the minimum AVR was observed among groups (median [quartile 1 (Q1); quartile 3 (Q3)]: 25 [0; 30]; 5 [−27; −75]; 10 [−6; −95]; 53 [19; 86]; 29 [5; 78]) for PHA, CKD, HTH, ISH, and healthy cats, respectively (*P* = .05). Basal serum aldosterone concentration (pmol/L) was significantly higher in PHA cats (median [Q1; Q3]: 2914 [2789; 4600]) than in CKD‐H cats (median [Q1; Q3]: 239 [189; 577], corrected *P* value = .003) and CKD‐NH cats (median [Q1; Q3]: 353 [136; 1371], corrected *P* value = .004).

**Conclusions and Clinical Importance:**

The oral telmisartan suppression test using a single dose of 2 mg/kg telmisartan did not discriminate cats with PHA from healthy middle‐aged cats or cats with diseases that may result in secondary hyperaldosteronism.

**Abbreviations**
ACTH: adrenocorticotropic hormone
ACVIM: American College of Veterinary Internal Medicine
Ao: aorta/aortic
ARR: aldosterone to renin ratio
AVR: aldosterone variation rate
CKD: chronic kidney disease
CKD‐H: hypertensive chronic kidney disease
CKD‐NH: non‐hypertensive chronic kidney disease
HCM: hypertrophic cardiomyopathy
HTH: hyperthyoidism
IRIS: International Renal Interest Society
ISH: idiopathic systemic hypertension
IVSd: end‐diastolic thickness of the interventricular septum
LA: left atrium/left atrial
LCMS/MS: liquid chromatography and tandem mass spectrometry
LFVWd: end diastolic thickened of the left ventricular free wall
LV: left ventricular
PHA: primary hyperaldosteronism
PRA: plasma renin activity
RAAS: renin‐angiotensin‐aldosterone system
SAS: Statistical Analysis System
SBP: systolic systemic arterial blood pressure
SF: shortening fraction
T4: serum thyroxin concentration
TOD: target organ damage
TST: telmisartan suppression test
UACR: urinary aldosterone‐to‐creatinine ratio
UPLC‐MS/MS: ultra‐performance liquid chromatography and tandem mass spectrometry

## 1 INTRODUCTION

Despite being increasingly recognized as the most common adrenocortical disorder in cats,^1^ definitive diagnosis of primary hyperaldosteronism (PHA) remains a challenge. The disease is defined by autonomous, excessive production of mineralocorticoids (mainly aldosterone) either as a consequence of an adrenal tumor or, presumably less frequently, of idiopathic micronodular hyperplasia in the adrenal cortex.^2^ One of the major difficulties in diagnosing this adrenal disease in cats is to differentiate PHA (“low‐renin” hyperaldosteronism) from secondary hyperaldosteronism (“high‐renin” hyperaldosteronism). The latter is characterized by the presence of an underlying disease (e.g., volume depletion, hyperthyroidism [HTH], or heart disease) that causes activation of the entire renin‐angiotensin system, resulting in aldosterone hypersecretion.^1^

Diagnostic criteria for PHA historically have relied on the detection of increased plasma or serum aldosterone concentration with consequently suppressed plasma renin activity (PRA) and an increased aldosterone‐to‐renin ratio (ARR).^2^, ^3^, ^4^ However, several limitations to this approach must be considered. First, assays for PRA are not widely available and require stringent sample handling because blood must be rapidly centrifugated, frozen, and transported on dry ice.^3^ Second, in human medicine, ARR is considered to be a screening tool with low specificity, and positive ARR results need further confirmation using dynamic endocrine testing.^5^ Third, the ability of the ARR to differentiate PHA from secondary hyperaldosteronism has not been thoroughly studied in cats. Therefore, development of a reliable and easily accessible test is needed for the diagnosis of PHA in cats.

Several exploratory tests have been developed to identify autonomous and excessive aldosterone production in PHA.^6^, ^7^, ^8^, ^9^, ^10^, ^11^, ^12^ For the diagnosis of PHA in humans, 4 testing procedures currently are recommended by the European Endocrine Society guidelines^13^: the fludrocortisone suppression test, the oral sodium loading test, the saline infusion test, and the captopril challenge test. To date, published evidence is insufficient to recommend 1 test over the others. In cats, oral fludrocortisone and oral sodium loading tests already have been evaluated.^8^, ^9^, ^10^ In 19 cats with systemic hypertension caused by PHA (n = 9) or other causes (n = 10), combined evaluation of basal urinary aldosterone‐to‐creatinine ratio (UACR) and UACR after PO fludrocortisone administration could not substantially discriminate between cats with and without PHA.^8^ Surprisingly, 4/9 cats with PHA showed UACR suppression, which may raise concerns about the interpretation of discordant results when performing such tests. Although the urinary fludrocortisone test is of undeniable interest, its disadvantages must be considered. It requires administration of fludrocortisone at home, or in‐hospital, twice a day for 4 days, which requires owner and animal compliance. It also entails giving mineralocorticoids to an animal that already suffers from an overproduction of these hormones. As a result, hypokalemia was observed in 7/19 treated cats, and in 1 of these muscle weakness occurred.

A recent study investigated a suppression test using PO telmisartan. It showed that PO administration of 2 mg/kg telmisartan suppressed plasma aldosterone concentration by a minimum of 33% in healthy young cats, but did not decrease aldosterone concentration significantly in cats with PHA; minimal overlap was observed between the 2 groups.^14^

Our aims were to extend the results of the previous study with further investigations of the accuracy of the telmisartan suppression test (TST) for PHA diagnosis in groups of cats with different clinical profiles. We hypothesized that telmisartan administration would suppress aldosterone secretion in healthy middle‐aged cats as well as in cats with secondary hyperaldosteronism, but not in cats with PHA. We also hypothesized that the TST would be safe and would not induce clinically relevant variations in blood pressure or serum potassium concentration. A secondary aim was to determine if hypertensive cats without adrenal masses, HTH or azotemia would have TST results consistent with occult PHA.

## 2 MATERIALS AND METHODS

Cats >5 years of age were prospectively recruited from cats visiting the veterinary teaching hospital of École Nationale Vétérinaire de Maisons Alfort between September 2019 and September 2021. Each owner gave informed consent for the cat's participation in the study. Baseline evaluations included routine measurements of hematological variables, biochemical analytes, serum thyroxine concentration (T4), and urinalysis. Urine was collected by ultrasound‐guided cystocentesis. Systolic systemic arterial blood pressure (SBP) was measured using the oscillometric method (PetMap Graphic II, Ramsey Medical), according to American College of Veterinary Internal Medicine (ACVIM) consensus guidelines^15^ by a single investigator (MK). Systemic arterial hypertension was defined as SBP ≥160 mmHg with evidence of target organ damage (TOD), or SBP ≥180 mmHg (with or without TOD). Hypertrophic cardiomyopathy (HCM) and retinopathy were considered as possible consequences of TOD.^15^ Fundus examination was performed after pupil dilatation using topical tropicamide. Fundus abnormalities considered to be consistent with hypertensive retinopathy included tortuous retinal vessels, preretinal or intraretinal hemorrhage, retinal edema, or retinal detachment.

Hypertrophic cardiomyopathy was defined as diffuse or regional increased left ventricular (LV) myocardium thickness with a nondilated LV chamber, according to ACVIM consensus guidelines.^16^ End‐diastolic thicknesses of the LV free wall (LVFWd) and the interventricular septum (IVSd) as well as LV end‐diastolic and end‐systolic internal diameters were measured using the 2‐dimensional (2D)‐guided M‐mode as recommended by the ACVIM consensus guidelines,^16^ and the LV shortening fraction (SF%) then was calculated. For each cat, all M‐mode measurements were compared with the 95% prediction intervals assessed according to body weight from a large population of healthy cats.^17^ Extreme LV hypertrophy was defined as LVFWd, IVSd, and subaortic IVSd ≥9 mm.^18^ The subaortic interventricular septal thickness also was measured at end‐diastole by 2D mode from the right parasternal 5‐chamber view at the mitral valve‐chordae tendineae interface as previously described and compared with reference ranges.^19^ The left atrial (LA) and aortic (Ao) diameters were measured at end‐diastole using a 2D method from the right parasternal short axis view, as previously described and the LA:Ao ratio then was calculated, with LA enlargement defined as an LA:Ao >1.2 (upper cutoff obtained from a population of 100 prospectively recruited healthy cats).^19^ Continuous‐wave Doppler recorded using the left apical 5‐chamber view was used to diagnose LV outflow tract obstruction defined as diffuse LV outflow tract turbulence and peak systolic outflow velocity ≥2.5 m/s.^20^ Additionally, each HCM phenotype was classified according to the staging system proposed by the ACVIM consensus statement on cardiomyopathies in cats.^15^ Adrenal glands were considered to be enlarged if cranial height was >4.5 mm or if caudal height was >4.8 mm.^21^

Echocardiography, fundic examination and abdominal ultrasonography were performed by a board‐certified specialist, resident, or trained clinician in the corresponding field.

To be eligible for the study, cats had to meet the clinical criteria for 1 of the 5 study groups. The PHA group consisted of cats with a diagnosis of PHA based on suggestive history, clinical signs, and biological findings combined with documentation of either an increased serum aldosterone concentration in the presence of hypokalemia without azotemia, or increased serum aldosterone concentration associated with an adrenal mass or bilateral adrenal hyperplasia. The CKD group consisted of cats with either hypertensive chronic kidney disease (CKD; CKD‐H) or non‐hypertensive CKD; (CKD‐NH), diagnosed according to the International Renal Interest Society (IRIS) guidelines.^22^ The HTH cats consisted of cats with HTH (based on increased serum total or free T4 concentrations). The idiopathic systemic hypertension (ISH) cats consisted of cats with idiopathic systemic arterial hypertension, which was defined as systemic arterial hypertension without an identified cause despite thorough investigations (specifically, exclusion of CKD, HTH, diabetes mellitus, hyperadrenocorticism, adrenal mass, and obesity). The last group (healthy cats) consisted of healthy, normotensive, client‐owned cats with no previously diagnosed conditions, no abnormalities detected by physical examination, no findings of concern in blood biochemistry and electrolyte analyses and with serum T4 concentration within the reference internal. Non‐inclusion criteria included anemia, receipt of any treatment that could interfere with the renin‐angiotensin‐aldosterone system (RAAS; e.g., amlodipine, spironolactone, angiotensin II receptor blockers, angiotensin converting enzyme inhibitors, sildenafil, pimobendan, methimazole or diuretics) within the previous 2 weeks, and heart failure.

The TST was performed as previously described, by the same investigator (MK).^14^ In brief, serum aldosterone concentration was measured before (T0), 1 hour after (T1), and 1.5 hours after (T1.5) PO administration of 2 mg/kg telmisartan (Semintra, Boehringer Ingelheim). Difficulty in administering the medication to each cat was evaluated subjectively on a scale from 0 (very easy) to 4 (impossible). Systolic blood pressure (SBP) and serum potassium concentration were measured at T0, T1, and T1.5 to evaluate safety. During the test, cats were kept in a quiet environment. Blood was collected from the jugular vein with cats positioned in sternal recumbency. After centrifugation, serum was stored at −80°C until batch analysis to measure aldosterone concentration by a radio‐immunoassay (RIAZENco Aldosterone, ZenTech). The assay was internally validated (intra‐ and inter‐assay coefficients of variation at 3 ranges of concentrations (90, 291, and 585 pmol/L) were between 1.9% to 4.5% and between 5.0% to 8.0%, respectively. Variations in serum aldosterone concentration were calculated as the difference in serum aldosterone concentration between T0 and both T1 and T1.5 (ΔAldo_T1_ and ΔAldo_T1.5_). Aldosterone variation rates (AVR) at T1 and T1.5 were calculated as (aldosterone_Tx_ − aldosterone_T0_)/aldosterone_T0_. The minimum change in serum aldosterone concentration (ΔAldo_min_) and the minimum AVR (AVR_min_) were determined across T1 and T1.5 values. The PRA was determined as the angiotensin I formation rate in plasma. An angiotensin I stabilizing inhibitor cocktail (Z‐Pro‐Prolinal, aminopeptidase inhibitor, EDTA, AEBSF buffered in phosphate‐buffered saline [pH 7.4]) was added to the samples before dividing the sample into 2 aliquots. While 1 aliquot remained on ice as a baseline control to quantify the amount of angiotensin I that was present at the beginning of the incubation period, the second aliquot was incubated at 37°C to quantify the amount of angiotensin I that was present after the incubation period. After incubation, angiotensin I was quantified using ultra‐performance liquid chromatography and tandem mass spectrometry (UPLC‐MS/MS) in both aliquots using stable isotope‐labeled internal standardization. The PRA was calculated from these 2 results by subtracting the baseline control result from the incubated sample result and the result was reported as angiotensin I in pmol/L/h. Serum telmisartan concentration was determined at T1.5 by liquid chromatography and tandem mass spectrometry (LC‐MS/MS).

All statistical analyses were performed using Statistical Analysis System (SAS) University Edition 9.04.01M6P11072018. Continuous variables are presented as medians (1st quartile [Q1] to 3rd quartile [Q3]). Binary variables were compared among groups using the *χ* ^2^ test or Fisher's exact test, depending on the size of the sample. If significant differences were observed, pairwise comparisons were conducted using the same tests and Holm's correction method was applied. Continuous variables were compared among groups using the Kruskal‐Wallis test. If significant differences were observed, pairwise comparisons were conducted using the Mann‐Whitney *U* test and Holm's correction method was applied. The Spearman correlation test was used to evaluate the association between serum telmisartan concentration and AVR at T1.5 and between PRA and AVR at T1.5. Statistical significance was set at *P* < .05.

## 3 RESULTS

Thirty‐eight cats were enrolled in the study and allocated to 1 of the 5 groups. Baseline demographic, clinical, and biochemical data are presented in Table 1. Additional characteristics of individual cats with PHA are described in Table S1. Serum potassium concentration was significantly lower in cats with PHA (2.4 mmol/L [Q1: 2.4; Q3: 2.8]) compared with other groups (4.1 mmol/L [Q1: 4.0; Q3: 4.7]; *P* = .01). Hypokalemia was detected in 4/5 cats with PHA, 2/7 cats with CKD‐NH, and in 2/9 cats with HTH.

**TABLE 1:** Baseline characteristics of the study populations (n = 38).

	PHA	CKD‐H	CKD‐NH	HTH	ISH	Healthy	Reference interval
n	5	9	7	9	2	6	
Age (years)	10 [10; 17]	15 [14; 17]	15 [8; 15]	16 [14; 17]	12 [9; 14]	8 [7; 9]	
Urea (mmol/L)	10.0 [6.5; 15.0]	15.3 [12.0; 21.2]	12.5 [12.0; 26.0]	13.0 [8.0; 14.7]	11.0 [4.20; 17.9]	9.5 [5.8; 9.9]	6.7‐13.4
Creatinine (μmol/L)	128 [90; 177]	186 [168; 305]	168 [151; 186]	96 [84; 124]	97 [80; 115]	89 [80; 97]	46‐142
Urine specific gravity	1.030 [1.026; 1.040]	1.016 [1.015; 1.025]	1.015 [1.012; 1.018]	1.028 [1.018; 1.031]	1.030 [1.025; 1.035]	1.016 [1.012; 1.020]	>1.035
Aldosterone (pmol/L)	2 914 [2.789‐4.600]	239 [189‐577]	353 [136‐1 371]	389 [264‐478]	344 [169‐519]	137 [128‐227]	14‐258
Plasma renin activity (pmol/L/h)	2123 [352; 10 377]	193 [160; 494]	2450 [778; 7080]	634 [575; 3296]	462 [351; 572]	578 [253; 906]	Not determined yet
T4 (nmol/L)	25 [6; 56]	27 [25; 36]	16 [6; 23]	108 [77; 176]	32 [27; 37]	28 [23; 31]	15‐50
Sodium (mmol/L)	149 [145; 154]	157 [154; 158]	149 [145; 156]	152 [152; 153]	158 [157; 159]	155 [154; 157]	150‐165
Potassium (mmol/L)	2.4 [2.4; 2.8]	4.1 [3.9; 4.7]	4.4 [3.5; 4.7]	4.1 [4.1; 4.6]	4.0 [3.9; 4]	4.2 [4.1; 4.6]	3.6‐5.5
Blood pressure (oscillometry, mmHg)	220 [210; 220]	220 [200; 230]	140 [135; 150]	160 [140; 190]	240 [220; 260]	138 [130; 140]	110‐160

*Note*: Results are presented as medians [1st quartile; 3rd quartile].

Abbreviations: CKD‐H and CKD‐NH, hypertensive and non‐hypertensive chronic kidney disease; HTH, hyperthyroidism; ISH, idiopathic systemic arterial hypertension; PHA, primary hyperaldosteronism.

The prevalence of systemic hypertension in PHA cats was not significantly different from that in CKD cats (*P* = .36) or HTH cats (*P* = .27; Table 2). In PHA cats, 4/5 had systemic hypertension, all with SBP >200 mmHg. In the fifth cat without detected systemic hypertension, SBP only was measured once, and it is possible that episodic systemic hypertension may have been missed. All cats with PHA showed signs of target organ damage (TOD): 2 had LV hypertrophy, and 4 had hypertensive retinopathy. In the CKD‐H group, 8/9 (89%) cats had SBP >200 mmHg and all 9 cats had evidence of TOD. In the HTH group, 3/9 cats (33%) were hypertensive, and of these, 2 had SBP >200 mmHg, 2 presented with concurrent azotemia, and 1 had evidence of HCM phenotype. Both of the cats with ISH were severely hypertensive (>220 mmHg) and had signs of hypertensive retinopathy. Only 1 of them presented with HCM phenotype.

**TABLE 2:** Blood pressure, echocardiographic and fundus examination findings in the study populations at presentation (baseline, n = 38).

	PHA	CKD‐H	CKD‐NH	HTH	ISH	Healthy
n	5	9	7	9	2	6
Systemic hypertension	4 (80%)	9 (100%)	0 (0%)	3 (33%)	2 (100%)	0 (0%)
Hypertensive retinopathy	4 (80%)	7 (78%)	0 (0%)	0 (0%)	2 (100%)	ND
Hypertrophic cardiomyopathy phenotype	2 (40%)	5 (56%)	2 (29%)	3 (33%)	1 (50%)	ND

Abbreviations: CKD‐H and CKD‐NH, hypertensive and non‐hypertensive chronic kidney disease; HTH, hyperthyroidism; ISH, idiopathic systemic arterial hypertension; ND, not determined; PHA, primary hyperaldosteronism.

The prevalence of hypertensive retinopathy in PHA group was not significantly different from that in the CKD‐H (*P* = .99) or ISH (*P* = .99) groups, but it was significantly higher than the prevalence in HTH group (*P* = .01; Table 2). Thirteen cats presented with HCM phenotype: 2 with PHA, 5 with CKD‐H, 2 with CKD‐NH, 3 with HTH, and 1 with ISH (Table 2). No significant difference was found in the prevalence of HCM between PHA groups and CKD, HTH and ISH (*P* = .76) groups. Median end‐diastolic LA:Ao ratio was 1.0 (Q1: 0.99; Q3: 1.13). The fractional shortening was normal in all cats with HCM phenotype (57%; [Q1: 50; Q3: 68]). Two cats had dynamic LV outflow tract obstruction: 1 with CKD‐NH and 1 with HTH with corresponding peak systolic pressure gradients of 205 and 37 mmHg, respectively. Among cats with HCM phenotype, 4 had hypertrophy of 1 myocardial segment alone: IVSd (n = 2), subaortic IVSd (n = 1), and LVFWd (n = 1). Two cats had hypertrophy of 2 myocardial segments (IVSd and LVFWd or subaortic IVSd), and 7 had hypertrophy of all 3 segments. Only 1 cat (with HTH) had severe myocardial hypertrophy, affecting the IVSd only. All HCM phenotypes were classified as stage B1 according to ACVIM classification guidelines, except for 2 cats (1 with CKD‐NH and 1 with HTH) that were classified as stage B2 because of gallop sound and severe LV hypertrophy, respectively.

**FIGURE 1:**
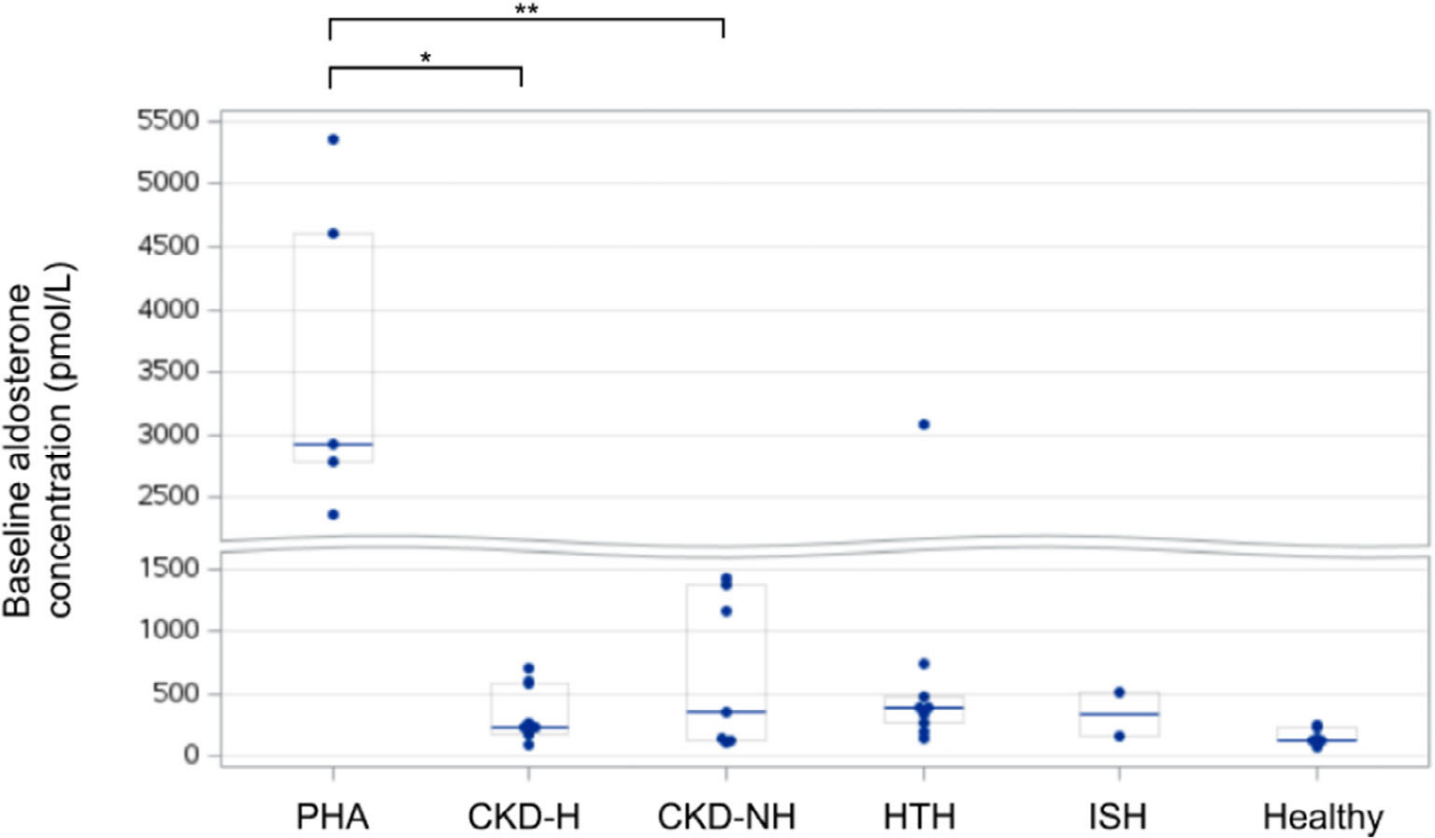
Baseline serum aldosterone concentration in each group. Asterisks (*, **) indicate significantly different values. Horizontal lines indicate the median values, and boxes delineate the 25th and 75th percentiles. Dots represent individual observations. CKD‐H and CKD‐NH, hypertensive and non‐hypertensive chronic kidney disease; HTH, hyperthyroidism; ISH, idiopathic systemic arterial hypertension; PHA, primary hyperaldosteronism.

Baseline serum aldosterone concentration (Figure 1) was significantly different among groups (*P* = .004). In pairwise comparisons, baseline aldosterone concentration was significantly higher in cats with PHA (2914 pmol/L; [Q1: 2789; Q3: 4600]) than in cats with CKD‐H (239 pmol/L; [Q1: 189; Q3: 577]; corrected *P* value = .003) and CKD‐NH cats (353 pmol/L; [Q1: 136; Q3: 1371]; corrected *P* value = .004). Other differences in serum aldosterone concentration between groups were not significant.

**FIGURE 2:**
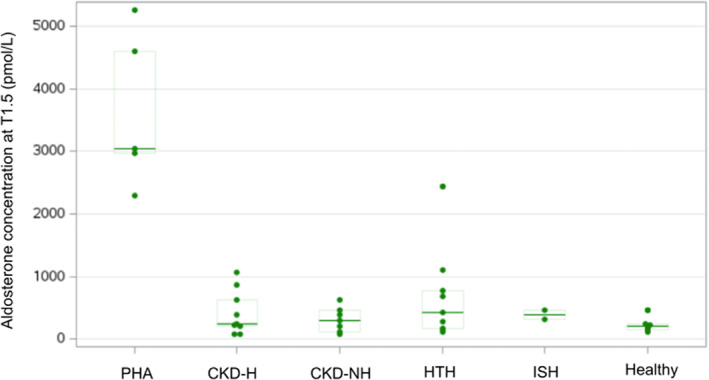
Serum aldosterone concentration at T1.5 in each group. See Figure 1 for legends.

**FIGURE 3:**
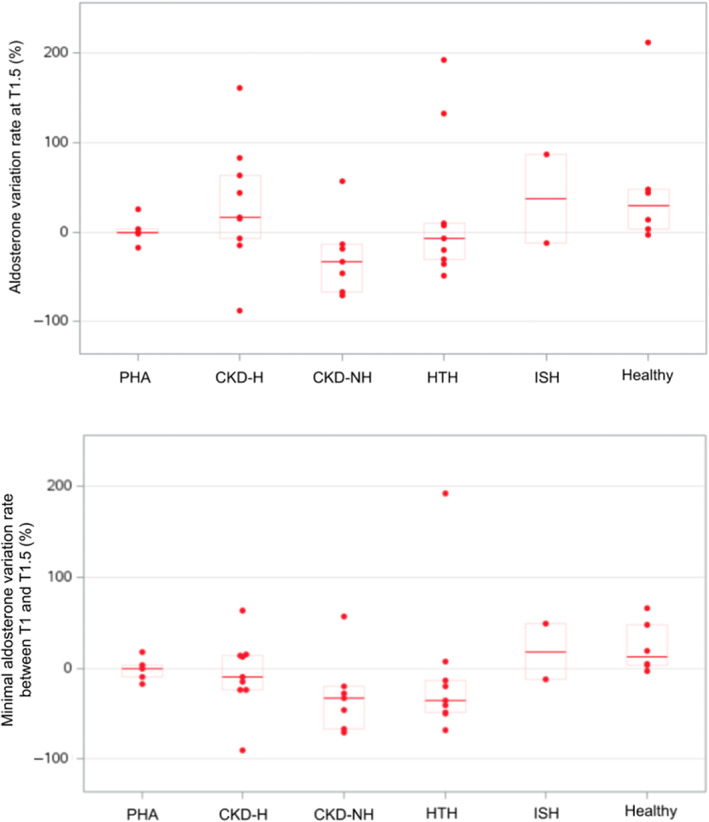
Aldosterone variation rate at T1.5 and minimal aldosterone variation rate between T1 and T1.5 in each group. See Figure 1 for legends.

**FIGURE 4:**
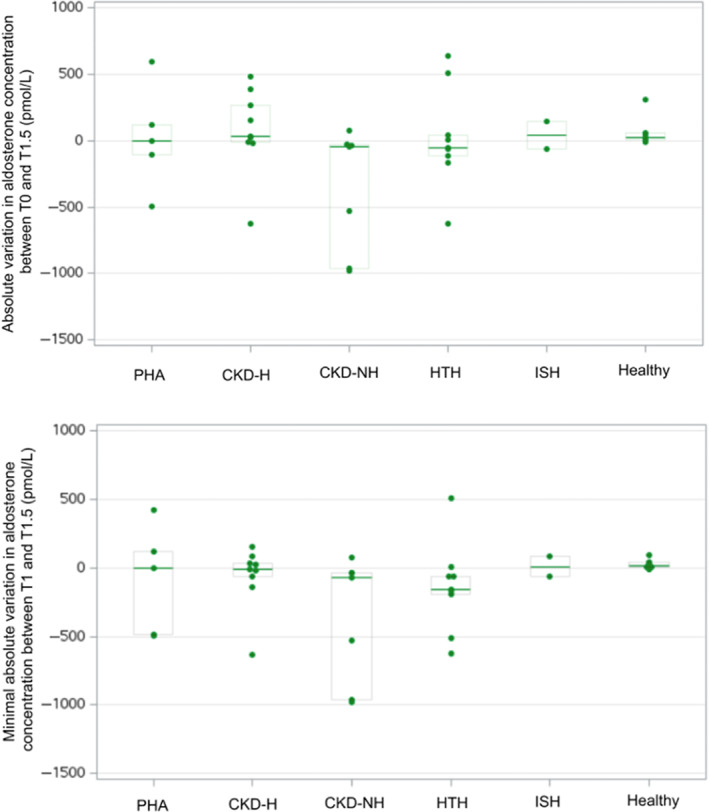
Absolute variation in aldosterone concentration between T0 and T1.5, and minimal absolute variation in aldosterone concentration between T1 and T1.5 in each group. See Figure 1 for legends.

The TST was well tolerated; none of the cats experienced hyperkalemia or systemic hypotension. Serum aldosterone concentrations at T1.5 for the groups are represented in Figure 2. No significant differences were found among groups in AVR_T1_ (*P* = .15), AVR_T1.5_ (*P* = .12; Figure 3), and AVR_min_ (*P* = .05; Figure 3). No significant differences were found among groups in ΔAldo_T1_ (*P* = .17), ΔAldo_T1.5_ (*P* = .24; Figure 4), and ΔAldo_min_ (*P* = .15; Figure 4). None of the healthy cats showed aldosterone suppression at either T1 or T1.5.

**FIGURE 5:**
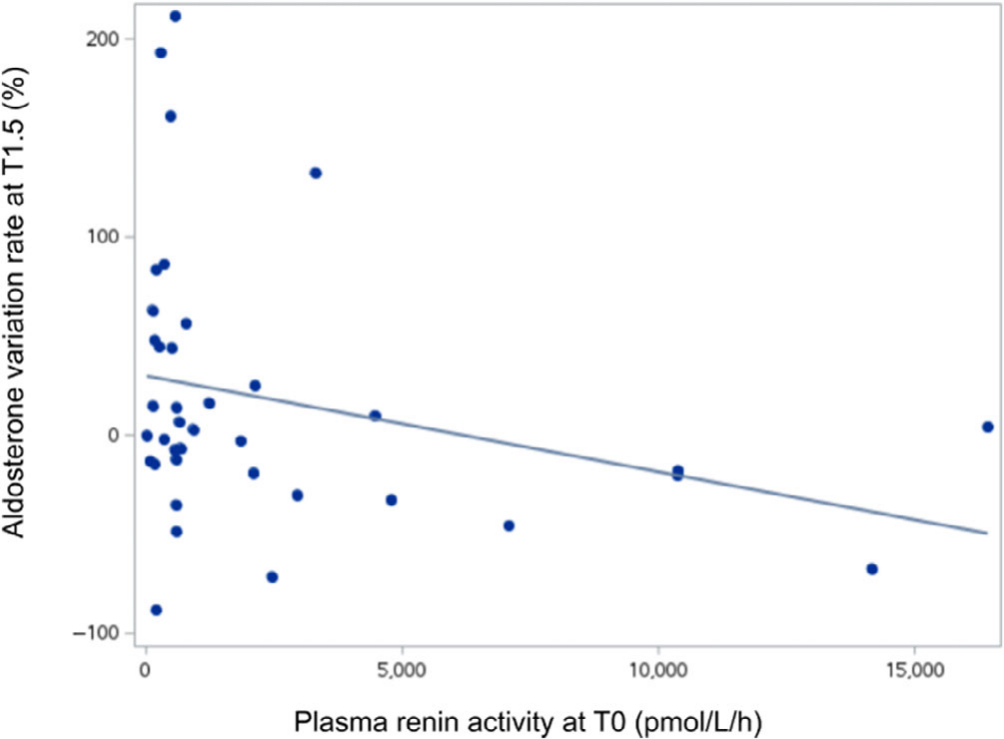
Aldosterone variation rate at T1.5 plotted against baseline plasma renin activity for the whole studied population.

Difficulty in administering telmisartan was scored as 0/4 in 29 cats (76%) and 1/4 in 9 cats (24%). Serum telmisartan concentration at T1.5 was 186 nmol/L (Q1: 93; Q3: 459); the data distribution was extremely wide, with concentrations ranging from 4 to 3397 nmol/L, and the coefficient of variation was 118%. Poor correlation was found between AVR_T1.5_ and serum telmisartan concentration at T1.5 (*r* = −.04; *P* = .80). Greater difficulty in administration of telmisartan was not associated with serum telmisartan concentration (*P* = .74). Moderate correlation was observed between AVR_T1.5_ and baseline PRA (*r* = −.28; *P* = .09; Figure 5).

## 4 DISCUSSION

We report investigation of an outpatient, dynamic, and short duration endocrine test for the diagnosis of PHA in cats. Unfortunately, the test investigated did not discriminate cats with PHA from other cats. The development of an accurate diagnostic test for PHA in cats is needed because definitive diagnosis of this disease is challenging and it may be underdiagnosed.^1^, ^2^ The clinical presentation of PHA usually lacks specificity because the condition shares clinical and biological features (e.g., systemic hypertension, hypokalemia) with other common diseases in geriatric cats, especially CKD and HTH. These diseases not only mimic PHA in clinical presentation, but they also confound diagnostic evaluation by causing activation of the RAAS.^23^, ^24^, ^25^ For these reasons, the greatest challenge in the diagnostic investigation of RAAS disorders is differentiation of PHA from aldosterone hypersecretion due to stimulation of the entire renin‐angiotensin‐aldosterone system caused by extra‐adrenal diseases (i.e., secondary hyperaldosteronism).

When trying to develop a confirmatory test for an endocrine disease characterized by hypersecretion of a hormone, it is natural to consider a suppression test. These tests aim to confirm that production of the hormone is not downregulated by an exogenous inhibitor, because it is being secreted autonomously.^8^, ^9^, ^10^ A suppression test using losartan, an angiotensin II receptor antagonist, has been evaluated in humans.^12^ The test is rapid and practical, requiring a single blood sample 2 hours after PO administration of losartan. Moreover, a study that included patients > 50 years of age suggested that the losartan suppression test was conservative than traditional exogenous mineralocorticoid‐based tests in patients with cardiac or renal impairment.^12^ Such comorbidities are thought to be frequent in cats with PHA.^1^, ^2^, ^4^ In veterinary medicine, telmisartan, another angiotensin receptor antagonist, is already available commercially (Semintra, Boehringer Ingelheim) and approved in cats for the management of proteinuria in CKD and systemic hypertension. In a pilot study, the TST accurately discriminated between 10 healthy cats and 6 cats with PHA.^14^ These preliminary data indicated that a dose of 2 mg/kg was more effective at differentiating healthy cats from cats with PHA than a dose of 1 mg/kg, and that both T1 and T1.5 sampling times were necessary.

In our study, although telmisartan did not significantly suppress serum aldosterone concentration in cats with PHA, the TST was unable to differentiate between these cats and cats with diseases that potentially result in secondary hyperaldosteronism (e.g., CKD, HTH). Furthermore, telmisartan did not suppress serum aldosterone concentration in any of the healthy cats. From our results, we could speculate that suppression of serum aldosterone concentration of at least 20% excludes PHA. However, our results contradict the results of the previous study, in which all healthy cats showed at least 33% aldosterone suppression.^14^ Because the main difference in the healthy cat population between the 2 studies was age (median 3 years in the pilot study vs 8 years in the our present study), we speculate that telmisartan has a different pharmacokinetic or pharmacodynamic profile in older cats, or that older cats are better able to resist its suppressive effects on aldosterone secretion. An “aldosterone breakthrough” effect is described in the human and veterinary medical literature,^26^, ^27^ when serum aldosterone concentrations do not remain suppressed despite ongoing treatment with an angiotensin converting enzyme inhibitor or angiotensin receptor antagonist; the serum concentration of circulating aldosterone may even increase above pretreatment concentrations. However, this escape phenomenon usually is reported after months of treatment in humans, and was detected after at least 7 days of telmisartan administration in dogs.^28^, ^29^ Because the cats in our study received a single dose of telmisartan, it is unlikely that they exhibited aldosterone breakthrough in the strictest sense. From a pharmacodynamic perspective, it seems reasonable to assume that homeostatic mechanisms in a healthy animal initially would compensate for a pharmacological perturbation, and that accordingly our cats may have failed to suppress aldosterone. It is possible that measuring serum aldosterone concentration after several days of telmisartan administration would lead to more marked aldosterone suppression, yielding more accurate results than the protocol we have used here. This approach was not taken because our aim was to develop a confirmatory test that is easier to perform than the fludrocortisone suppression test.

Another explanation for our unexpected results may be related to variability in the pharmacokinetics of telmisartan. We used sampling times for aldosterone (T1 and T1.5) on the basis of the pilot study,^14^ which was itself informed by pharmacokinetic data for telmisartan in 12 young laboratory cats. Our current results, however, have identified unexpected high variability in serum telmisartan concentration at T1.5. Moderate variability in maximal serum telmisartan concentration (*C*_max_) also was observed in the Semintra licensing study (coefficient of variation between 33% and 45%) but was lower than in our population (118%).^30^ The *C*_max_ in the licensing study (between 377 and 717 pmol/L, reached 15‐23 minutes after PO administration) was, however, of the same order of magnitude as the median telmisartan concentration measured at T1.5 in our population (186 nmol/L; [Q1: 93; Q3: 459]). Thus, the pharmacokinetics of telmisartan could be different in cats that are older, or suffer from concomitant diseases, or both. In particular, more variation in bioavailability may explain the disparity of response to the TST in our population.^30^ That being said, we compared the aldosterone suppression between cats that had a telmisartan concentration at T1.5 >500 nmol/L and <500 nmol/L and the outcome of our study was not changed (median minimal AVR of 1%; [Q1: −71, Q3: 49] and −20%; [Q1: −90, Q3: 193], respectively). However, the fact remains that biological availability of telmisartan may be very poor or unpredictable after PO administration in middle‐aged cats, and that possibility should be taken into account when treating a proteinuric or hypertensive cat with telmisartan. As with other drugs, measurement of serum telmisartan concentration could become necessary for therapeutic adjustment.

Alternatively, the plasma half‐life of aldosterone might be longer than the 20 minutes we estimated. To our knowledge, the plasma half‐life of aldosterone in cats has not been established. If so, measuring aldosterone concentration >1.5 hours after telmisartan administration could be considered, as well as quantification of urinary aldosterone, which has been done in fludrocortisone suppression test studies. However, previous studies showed that urinary measurement of aldosterone may not be optimal^9^ because cats excrete very little aldosterone in urine compared to other species.^31^ Our results call for a better understanding of the pharmacokinetic and pharmacodynamic properties of telmisartan in cats, as well as the factors modifying its ability to suppress the RAAS. In particular, it is possible that the resistance to suppress aldosterone secretion is caused by neuropsychological or cardiovascular factors stimulating aldosterone secretion such as stress or hypovolemia. Last, we could postulate that the stress induced by blood sampling and hospitalization and resulting in acute changes of circulating adrenocorticotropic hormone (ACTH) concentration may have resulted in stimulation of aldosterone secretion, which overwhelmed angiotensin 2 receptor blockade. Given these considerations, although the TST as we performed it was not successful, it is possible that different doses or different sampling times could improve the test's performance.

In our study, cats were included in the PHA group without reference to renin activity. It could be argued that doing so decreased the accuracy of group allocation, but we do not believe that it necessarily calls into question the results. Cats with PHA presented with clinical and laboratory findings that were strongly suggestive of PHA. Likewise, we consider it unlikely that a substantial number of cats from the other groups had occult PHA, despite the possibility of occasional misdiagnosis. A test for ARR frequently has been suggested for confirmation of PHA in cats.^3^, ^4^ This test is based on the demonstration of low PRA in the face of high serum aldosterone concentration, indicating autonomous and dysregulated aldosterone synthesis. The test is not widely available in many parts of Europe. Moreover, in human medicine, ARR and PRA tests are used for screening only,^3^, ^5^, ^32^, ^33^ and the recommendation is to confirm a positive result for PHA with a dynamic test.^13^ In veterinary medicine, most studies investigating PHA in cats have used the ARR as the gold standard for diagnosis and comparison with other tests.^8^, ^10^ However, reference intervals usually are established in healthy animals of various age, but generally younger than the target population,^3^ and thresholds may not be applicable to a population of geriatric animals with frequent RAAS activation. Moreover, the same study^3^ showed that PRA decreased with age and was significantly higher in cats <5 years of age.^3^ Limited data is available on the ability of ARR ability to discriminate PHA from secondary hyperaldosteronism. Furthermore, PHA and secondary hyperaldosteronism may coexist.^4^ It also has been shown that PHA in cats caused by micronodular hyperplasia often is associated with normal ARR,^4^ which emphasizes the need for development of confirmation tests for PHA. Interestingly, our cats with presumed PHA that also had comorbidities potentially associated with secondary hyperaldosteronism (e.g., HTH, CKD, dehydration) presented with extremely high PRA, whereas the lowest PRA results were observed in the 2 cats that had normal hydration status on presentation (1 of them presenting with mild hyperthyroxinemia). Acknowledging the small number of observations and the possibility of misclassification, we hypothesize that in some cases of PHA, PRA may not be suppressed and thus may yield ARR results similar to those of cats with secondary hyperaldosteronism.^4^

Our study had some limitations. Baseline serum aldosterone concentration was extremely high in the cats classified as having PHA, and thus it is reasonable to question whether a confirmatory diagnostic test was relevant in the study population. In addition, the group allocation was not infallible because a gold standard diagnostic test was not used, because such a test does not yet exist in veterinary medicine. Notwithstanding the possibility of classification bias, our main finding was that neither healthy cats nor cats with diseases that may result in secondary hyperaldosteronism showed significant suppression of serum aldosterone secretion during the TST. Thus, although it is unlikely that the TST we used could be used as a diagnostic tool for cats with PHA, other protocols might still be of interest. A dose‐finding study is needed in middle‐aged and older cats to determine the required dosage of telmisartan to suppress aldosterone secretion. Furthermore, more prolonged treatment with telmisartan may prove to be helpful, as has been shown to be necessary for fludrocortisone acetate. Echocardiography, fundic examination, and abdominal ultrasonography were performed by clinicians with different levels of experience, which may have led to interindividual variation in the results reported. Lastly, the secondary aim of our study was to determine if hypertensive cats without adrenal masses, HTH or azotemia had TST results consistent with occult PHA but we could include only 2 cats in this group over the study period, resulting in a lack of statistical power.

## 5 CONCLUSIONS

The TST based on measurement of serum aldosterone concentration before, and 1 and 1.5 hours after PO administration of 2 mg/kg telmisartan did not differentiate between healthy mature cats and cats with PHA or cats with diseases that may result in secondary hyperaldosteronism. The AVR at T1 and T1.5 were not significantly different between groups, and marked overlap was observed. No suppression of aldosterone secretion was observed in healthy cats and cats with diseases that may result in secondary hyperaldosteronism, which is contrary to our hypothesis. Further evaluation of our protocol for a TST to diagnose cats with PHA is not warranted. Development of an accurate confirmatory test for this disease will be a challenge for the coming years, because adrenal disorders in cats may be underdiagnosed and poorly understood and may play a pivotal role in the genesis of CKD or so‐called essential (or idiopathic) hypertension in cats. Data on RAAS modulation by telmisartan still is limited in cats. Future improvements in this field could result in development of a modified TST protocol with better diagnostic accuracy for PHA in cats than that described here.

## CONFLICT OF INTEREST DECLARATION

Residency program of Maxime Kurtz has received financial support by Royal Canin. No other authors have a conflict of interest.

## OFF‐LABEL ANTIMICROBIAL DECLARATION

Authors declare no off‐label use of antimicrobials.

## INSTITUTIONAL ANIMAL CARE AND USE COMMITTEE (IACUC) OR OTHER APPROVAL DECLARATION

The work described in this manuscript involved the use of non‐experimental (owned or unowned) animals and procedures that differed from established internationally recognized high standards (“best practice”) of veterinary clinical care for the individual patient. The study had prior ethical approval from the Comité d'éthique en Recherche Clinique (ComERC) from the Ecole nationale vétérinaire d'Alfort, Number 2020‐05‐24‐1. Written informed consent was obtained from the owner or legal custodian of all animals described in this work for all procedures undertaken.

## HUMAN ETHICS APPROVAL DECLARATION

Authors declare human ethics approval was not needed for this study.

## Supporting information


**Table S1.** Epidemiological, clinic‐pathological, medical imaging, and outcome data in the group of cats presenting with primary hyperaldosteronism (n = 5). BCS, body condition score; CKD, chronic kidney disease; DSH, domestic shorthair; HTH, hyperthyroidism; ND, not determined; NF, neutered female; NM, neutered male; RI, reference interval; SBP, systolic blood pressure (oscillometry, mmHg); SHA, secondary hyperaldosteronism; US, ultrasonography; USG, urine specific gravity.
