# Supplementary material for: Prospective evaluation of a telmisartan suppression test as a diagnostic tool for primary hyperaldosteronism in cats
Source: J Vet Intern Med. 2023 May 29;37(4):1348–57. doi: 10.1111/jvim.16741 (PMC10365049; doi:10.1111/jvim.16741)
Supplement: Supplementary file 1 — Table S1. Epidemiological, clinic‐pathological, medical imaging, and outcome data in the group of cats presenting with primary hyperaldosteronism (n = 5). BCS, body condition score; CKD, chronic kidney disease; DSH, domestic shorthair; HTH, hyperthyroidism; ND, not determined; NF, neutered female; NM, neutered male; RI, reference interval; SBP, systolic blood pressure (oscillometry, mmHg); SHA, secondary hyperaldosteronism; US, ultrasonography; USG, urine specific gravity. [file JVIM-37-1348-s001.pdf]

Case	Breed	Sex	Age (years)	BCS (/9)	Chief concern	Clinical dehydration or hypovolemia	Urea (mmol/L)	Creatinine (µmol/L)	T4 (nmol/L)	Sodium (mmol/L)	Potassium (mmol/L)	USG	SBP	Hypertensive retinopathy	Hypertrophic cardiomyopathy phenotype	Adrenal US findings	Aldosterone (pmol/L)	PRA (pmol/L/H)	Identified intercurrent disease causing possible SHA	Outcome
**RI**							6.7 - 13.4	46 - 142	15 - 50	150 - 165	3.6 - 5.5	> 1.035	110 - 160				14 - 258	ND		
**1**	DSH	NM	10	7	Anorexia for 1 week, weight loss	Yes	10	177	6.4	145	2.4	1.04	240	Yes	Yes	Left adrenal mass (31 mm)	2 365	2 123	CKD	Initial clinical improvement, euthanasia 4 months after initial presentation due to worsening clinical condition
**2**	DSH	NM	17	3	Anorexia, vomiting, progressive lethargy for several weeks	Yes	19	206	154	176	2.4	1.018	220	Yes	No	Right adrenal mass (6 mm)	2 914	16 422	CKD, HTH	Euthanasia following presentation, no necropsy was allowed
**3**	DSH	NM	9	4	Dysorexia for 12 days	No	6	81	56	144	2.1	1.03	135	No	Yes	Right adrenal mass (20 mm)	4 600	10	HTH	Euthanasia. Necropsy and adrenal histology consistent with adrenocortical carcinoma. Concurrent digestive toxoplasmosis
**4**	DSH	NF	10	7	Acute respiratory distress after 1 episode of	No	7	90	25	154	2.8	1.04	210	Yes	No	Right adrenal mass (38 mm)	5 362	352		Hemoabdomen following adrenalectomy, subsequent death
**5**	DSH	NF	17	2	Acute blindness for 24h, dysorexia for 2 days	Yes	15	128	6.4	149	4.3	1.026	220	Yes	No	Bilateral adrenal hyperplasia (7.1 and 6.3 mm)	2 789	10 377	Mild signs of CKD on US examination	Acute renal insufficiency 2 weeks following initial presentation, euthanasia
